# In preschool children, physical activity during school time can significantly increase by intensifying locomotor activities during physical education classes

**DOI:** 10.1186/s13104-018-3536-x

**Published:** 2018-07-03

**Authors:** Juliana Kain, Bárbara Leyton, Johana Soto-Sánchez, Fernando Concha

**Affiliations:** 1 Institute of Nutrition and Food Technology (INTA), University of Chile, Av El Líbano 5524, Santiago, Macul Chile; 2 University of Playa Ancha, Av Playa Ancha 850, Valparaíso, Chile

**Keywords:** Physical education lessons, Preschool children, MVPA, Motor skills, Accelerometer

## Abstract

**Objectives:**

After categorizing preschool children into “active and low active” according to their moderate and vigorous physical activity (MVPA) in PE classes (PE), we compared these two groups within each sex and by sex in: (a) % MVPA and MVPA minutes accrued from each fundamental motor skill (FMS) during PE and (b) % MVPA during school time.

**Results:**

532 children (mean age 5.2 years, 50% girls) were selected from a nationwide program which provides 3 weekly PE. Children wore accelerometers during one school day which included PE. We recorded the type and duration of each activity indicated by the teacher, classifying each one into the corresponding FMS, extracting its MVPA minutes from the accelerometer software. Children were categorized into active and low active. Comparisons used T-tests. In PE, active children accumulate 40 and 36 percentage points (pp) more MVPA minutes (boys and girls respectively), while during school time, 4 pp more in each sex. Girls are significantly less active. Just considering locomotion, active boys and girls accumulate 11 more MVPA minutes during PE. Active boys surpass the MVPA guideline for PE, while active girls almost reach it. Low active children (especially girls) should intensify locomotor activities during PE.

## Introduction

It has been shown that physical activity (PA) in preschool settings is associated with children’s daily PA. Within school time, the physical education lesson (PE) has been identified as the most important segment to intervene, as it is the only curricular subject to provide PA to all the children [1].

Fundamental motor skills (FMS) can be defined as “an organized series of basic movements which include a combination of movement patterns of body segments [2]. FMS categories include locomotion (e.g. running, hoping), object control (e.g. throwing and catching), fine motor skills (e.g. cutting) and balance (e.g. standing in one leg). Although it has been shown that FMS proficiency and physical activity (PA) are associated, this relationship among preschool children is conflicting. This could be due to the different methods used to determine both FMS proficiency and PA and/or that at this age, children have not yet mastered FMS as suggested by De Meester et al. [3] who showed that this association is weak at a young age, however it strengthens as children age. Although this might be the case, it is of utmost importance to improve FMS in preschool children in order to participate successfully in recreational activities as well as sports [4], considering also that motor skills track through childhood to adolescence [5]. One of the most effective ways to achieve proficiency in FMS is by increasing daily PA. Schools are the most important venues in promoting PA [6, 7] and within school time, PE classes contribute significantly not only to school’s moderate and vigorous PA (MVPA), but also to daily MVPA [8].

MVPA accumulated during PE is low. A systematic review [9] including studies published between 2005 and 2014 found that on average, the proportion of MVPA children accrue during PE classes was 32.6%. Although the recommendation that 50% of class time be spent on MVPA [10] is normally not attained, MVPA during PE has been shown to be higher than that accumulated during other segments of the school day [11], and that teacher-led PE lessons are one of the most effective ways to achieve this guideline [9].

Gender differences in MVPA among preschool children have been observed in several studies. For example, in Hong Kong [12] boys accumulated 42% MVPA during PE classes, versus 27.8% in girls. Among Brazilian elementary school children boys engaged in 44.1% and girls 21% MVPA during PE classes [13].

PE for preschool children in which children engage in sufficient MVPA should be structured in terms of the skills addressed. As reported by Morgan et al. [14] quality of instruction and time spent in practice are very important in improving FMS competence. Although FMS proficiency is normally determined by different field tests and results are obtained as scores for some or all motor skills, we consider that information regarding MVPA minutes accumulated from each FMS by preschool children who engage in sufficient compared to insufficient PA, is useful to know which is the most effective way to improve MVPA among inactive children. So, the purpose of this study is to compare active versus low active preschool children within each sex and by sex in (a) % MVPA and MVPA minutes accrued from each FMS during PE and (b) % MVPA during school time.

The Ethics Committee for Human Studies of the Institute of Nutrition and Food Technology (INTA), University of Chile approved this study. In addition, a signed informed consent form was obtained from a parent/guardian of every child.

## Main text

### Methods

The Chilean Ministry of Sports is implementing since 2014 a nationwide program to increase PA among low-income preschool children called “Active Preschools” or Jardín Activo [15]. It provides funding for certified physical education (PE) teachers to lead three structured PE lessons (PE) per week for about 7 months of the school year. Training of PE teachers is done through an on- line session of 1 h with the general objective of “strengthening basic motor skills, using active play while addressing gender differences”.

### Participants

In 2015, there were 311 JA programs in 92 preschools. We selected children from 66 preschools as described previously [16]. Briefly, in each school, the study PE teacher selected 4–5 preschool children (mean age 5.2 years) upon arrival (at least half of each gender), The children were equipped with an Actigraph GT3× accelerometers (Actigraph LLC, Pensacola, Florida, USA) which was removed when the school day ended. Accelerometers were programmed to record at a 15-s epoch length, which has been used in most studies including preschool children [17]. PA intensity was defined with Butte et al. cut-points [18]. During each lesson, the study PE teacher recorded the type and duration in minutes of each activity indicated to the children by the teacher hired by the Jardín Activo program. We then classified each activity into the different FMS: locomotion, object control, balance, combination of locomotion (locomotion plus object control and locomotion plus balance) and also “none” (defined as time when instructions could not be related to a FMS); for example, sit down and listen. Using the Acti Life 6 Software we extracted the MVPA data corresponding to each FMS during the PE lesson for the analyses.

During the study period, 164 PE were monitored. Because most lessons had a scheduled duration of 60 min (76.2%), the study only considered these for the analyses. The number of children with accelerometer data was 532. In addition, the different types of FMS boys and girls engage in during PE are shown as MVPA minutes accumulated from each one of them. According to the MVPA level in PE, we categorized the children into (a) “active”, defined as those who accumulated a total ≥ Percentile 75 (P 75) MVPA and (b) “low active” those who accrued < Percentile 25 (P 25) MVPA minutes. Both groups of children were selected according to the MVPA accumulated during one PE lesson throughout the 10 weeks of the study; each child participated in only one lesson. Because boys are more active than girls, cut points for P 25 and P 75 MVPA minutes were different by sex; 10.6 and 22.8 min in boys and 8.1 and 19.5 min in girls, respectively.

### Statistical analyses

Although in this study, we considered PE with a scheduled period of 60 min, the analyses based on the average actual time children participated (45 min). As stated above, we extracted MVPA minutes of each FMS recorded by us during PE. We then classified each activity into the corresponding FMS and extracted its MVPA minutes from the child’s accelerometer data.

We used t-tests to compare % MVPA accumulated by the very active versus low active children in PE lessons and during school time within each sex and by sex and MVPA minutes accumulated from each FMS during PE between these 2 groups, in each sex and by sex. All comparisons considered independent groups resulting in an average number for each variable per group.

### Results

**Fig. 1:**
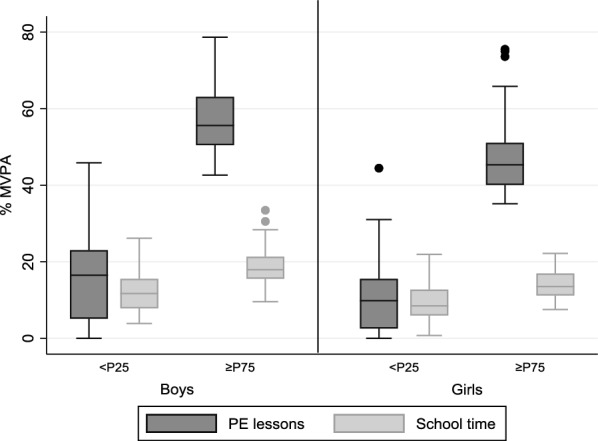
MVPA accumulated by “active versus low active” preschool children in PE and during school time, by sex (% and SD)

Figure 1 shows the difference in mean % MVPA accumulated by active versus low active boys and girls in PE and during school time. In both boys and girls, there is a significant difference between these 2 groups (40 and 36% points for boys and girls respectively). On average, active boys surpass the guideline for a good quality PE class, while girls almost reach it (47% MVPA). During school time, active children also accumulate significantly more % MVPA, approximately 4% points in each sex. Girls are significantly less active both in PE as during the entire school day.

In Table 1 we show the comparison of average MVPA minutes from each FMS accumulated by each group in PE. Active children accumulate significantly more MVPA minutes from all FMS except for locomotion combined in boys and object control in girls. In PE, active boys and girls accumulate approximately 11 more minutes in MVPA, only considering locomotion. In object control the difference is not negligible, especially in boys (3 more MVPA minutes).

**Table 1:** MVPA accumulated from the different FMS during PE by active versus low active preschool children (mean and SD)

MVPA from different FMS in PE (min)	Boys		Girls	
	Low active < P25	Active ≥ P75	Low active < P25	Active ≥ P75
Locomotion	2.91 (4.4)^1^	13.8 (8.6)^1^	1.87 (2.8)^1^	12.9 (9)^1^
Locomotion combined	3.04 (2.7)	4.3 (2.9)	1.0 (2)	3.1 (2.6)^1^
Object control	1.02 (2.2)^1^	4.9 (6.7)^1,2^	1.4 (2.9)	2.2 (4.8)^2^
Balance	0.14 (0.7)^1^	1.06 (3.6)^1^	0.06 (0.3)^1^	1.6 (4)^1^
None	0.4 (1)^1^	1.5 (0.8)^1^	0.62 (0.8)^1^	1.4 (3.9)^1^

^1^Significant difference between active versus low active children within each sex (p < 0.05)

^2^Significant difference between active children by sex (p < 0.05)

### Discussion

The most important results of this study show that in PE taught by specialists who give the same instructions as to the type and length of each activity, there is a marked difference in intensity in which 5-year-old children perform each FMS, especially in locomotor activities. Active boys, on average, surpass the MVPA guideline (50% MVPA during PE), while girls almost reach it (47%).

Even though MVPA accumulated from the different FMS is by definition not comparable, it is possible to compare the amount accrued from each FMS between more active versus low active children, within each sex and between boys and girls. This study shows that to increase intensity during PE, it is highly effective to consider firstly locomotor activities in both sexes. This will not only improve the quality of PE, but will also increase activity during school time, especially in boys.

Weaver et al. in a similar study [11] examined in 323 children from 7 schools (1st to 3rd grade) PA across the school day by segments. The authors assessed differences in children’s accumulation of MVPA in each school segment by classifying children as high or low active during school time. In that study, PE was taught on average for 54.6 min; % MVPA was 36.3 and 11 in high active versus low active boys, while in girls, these % were 31.3 and 10.6 respectively. When the authors considered MVPA accumulated during school time, these % were 12.8 and 4.2% for high and low active boys and 11.3 and 3.6 for girls respectively.

MVPA accumulated by the children in our study was significantly higher, both during PE as across the entire school day. Some possible explanations include that in our study all PE classes were taught by certified teachers in contrast to 4 out of 7 schools in the Weaver’s et al. study and also in our study children were younger (mean age, 5.2 versus 7.5 years) and the evidence shows that younger children are more active [19].

Several authors have reported that there is a positive association between motor skill scores and PA levels. Fisher et al. [20], noted in 4 years olds that more proficient children accumulated more MVPA while Williams et al. [21], found that preschool children in the highest tertile of motor scores, accumulated significantly more MVPA; this relationship was stronger between locomotor skills and PA. Foweather et al. [22], examined the association between FMS competence and daily PA among 3–5 years old children, showing that locomotor proficiency was positively associated with daily MVPA. Because in preschool children, FMS are still developing [23], scores may not reflect show proficiency accurately, so increasing intensity, especially in locomotion, which in turn is associated with more PA, will likely improve FMS scores in low active children.

Brown et al. [24] point out that young children should engage in “gender neutral activities” with teacher support, stressing the need for “teacher involvement to enhance PA”. In our study however, instructions during PE were not specifically directed at boys and were the same for all the children, while the amount of physical activity was significantly different within the same sex and by sex. This is probably due to the association between percent time in MVPA and motor skill score which is gender dependent as reported by Fisher et al. [20], and Cliff et al. [25].

The most important strength of this study is the use of an objective measurement of PA on a relatively large sample of 5-year-olds during school time and being able to extract the data corresponding to PE, in order to determine PA intensity and duration of each activity performed during the class.

### Conclusion

This study shows that a very effective way of reaching both the % MVPA guideline in PE as well as increasing MVPA during school time, is encouraging less active children to increase intensity (especially girls) when doing activities related to locomotion, such as jumping, leaping, hopping, running during PE. Achieving competence in motor skills should be a priority to increase MVPA in both sexes, encouraging teachers to identify different options to do so.

## Limitations

We have to recognize that this descriptive observational study has several limitations. These include not knowing if there were differences in teachers` previous training, attitudes, motivations or any other aspect of their behavior, all of which have been shown to influence how children perform in PE [26]. In addition, there were measurements that we were unable to collect for reasons beyond our control such as individual FMS scores, so as to assess if there is an association between them and MVPA minutes and the nutritional status of the children, in order to adjust % MVPA during PE and school time as well as intensity of each FMS by individual BMI Z score because it has been shown that excess weight has been associated with lower PA levels [27].

## Abbreviations

PA: physical activity
MVPA: moderate and vigorous physical activity
PE: physical education
FMS: fundamental motor skills
P: percentile
